# Epidermal Growth Factor Receptor (EGFR) Downregulation by Cetuximab in Salivary Duct Carcinoma: A Case Report

**DOI:** 10.7759/cureus.83763

**Published:** 2025-05-08

**Authors:** Hikari Saito, Takumi Kumai, Michihisa Kono, Kenzo Ohara, Miki Takahara

**Affiliations:** 1 Department of Otolaryngology-Head and Neck Surgery, Asahikawa Medical University, Asahikawa, JPN

**Keywords:** cetuximab, egfr, metastasectomy, salivary duct carcinoma, salivary gland cancer

## Abstract

Salivary duct carcinoma (SDC) is a high-grade salivary gland cancer with a poor prognosis. Although therapeutic antibodies capable of targeting several proteins expressed in SDC, such as epidermal growth factor receptor (EGFR) and human epidermal growth factor receptor 2 (HER2), have been developed, the stability of these antigens during recurrence remains unknown. Herein, we report a case of SDC in the submandibular gland wherein EGFR loss was pathologically confirmed after EGFR-targeted therapy. A 53-year-old man with a right submandibular mass underwent primary tumor resection with ipsilateral radical neck dissection and was diagnosed with SDC along with high EGFR and HER2 expression. Multiple pulmonary metastases appeared five months after the surgery, and the patient received 12 cycles of chemotherapy with paclitaxel and cetuximab. Although most metastatic nodules disappeared with the chemotherapy, a single nodule enlarged 15 months after the treatment. The resection of the pulmonary nodule pathologically confirmed the metastasis of SDC, wherein EGFR expression was downregulated. The patient has remained in remission for seven years after pulmonary metastasectomy without any additional treatment. As selective pressures caused by molecule-targeted therapies may downregulate the expression of the corresponding molecules, pathological examination using the latest tissue samples is ideal for elucidating the biological features of recurrent SDC. Our findings provide valuable insights for developing effective strategies for the treatment of cases with SDC recurrence post molecule-targeted therapy.

## Introduction

Salivary duct carcinoma (SDC) is a high-grade salivary gland cancer characterized by local aggressiveness and high rates of early recurrence and distant metastases [1]. Although the prognosis of SDC is poor, the identification of targetable molecules has helped in improving patient survival. Among salivary gland cancers, the expression of epidermal growth factor receptor (EGFR), human epidermal growth factor receptor 2 (HER2), and androgen receptor (AR) is unique to SDC. Clinical trials have shown that an anti-HER2 antibody (trastuzumab) therapy combined with chemotherapy or androgen deprivation therapy exhibits clinical benefits in SDC [2,3].

EGFR is widely expressed in salivary gland cancers, regardless of the type of tumor [4]. Specifically, it has been reported to be expressed in 24-55% of cases of SDC [4,5], which is comparable to that of HER2 [6]. Trastuzumab is an IgG1 antibody against HER2 that exhibits antitumor activity via antibody-dependent cytotoxicity (ADCC). Cetuximab, an IgG1 antibody against EGFR, also exhibits ADCC [7]; therefore, its use can help achieve clinical benefits in SDC treatment. According to literature, cetuximab-based therapy is effective in patients with SDC [8,9]. The drawback of targeting a specific protein is that the tumor may escape molecule-targeting therapy by attenuating the expression of the corresponding protein during recurrence. 

Here, we report a case of SDC in the submandibular gland wherein cetuximab-based chemotherapy helped diminish most of the metastatic pulmonary nodules, and the expression of EGFR in a cetuximab-resistant nodule was considerably downregulated. Our findings provide valuable insights for developing effective strategies for the treatment of cases with SDC recurrence post molecule-targeted therapy.

## Case presentation

**Figure 1:**
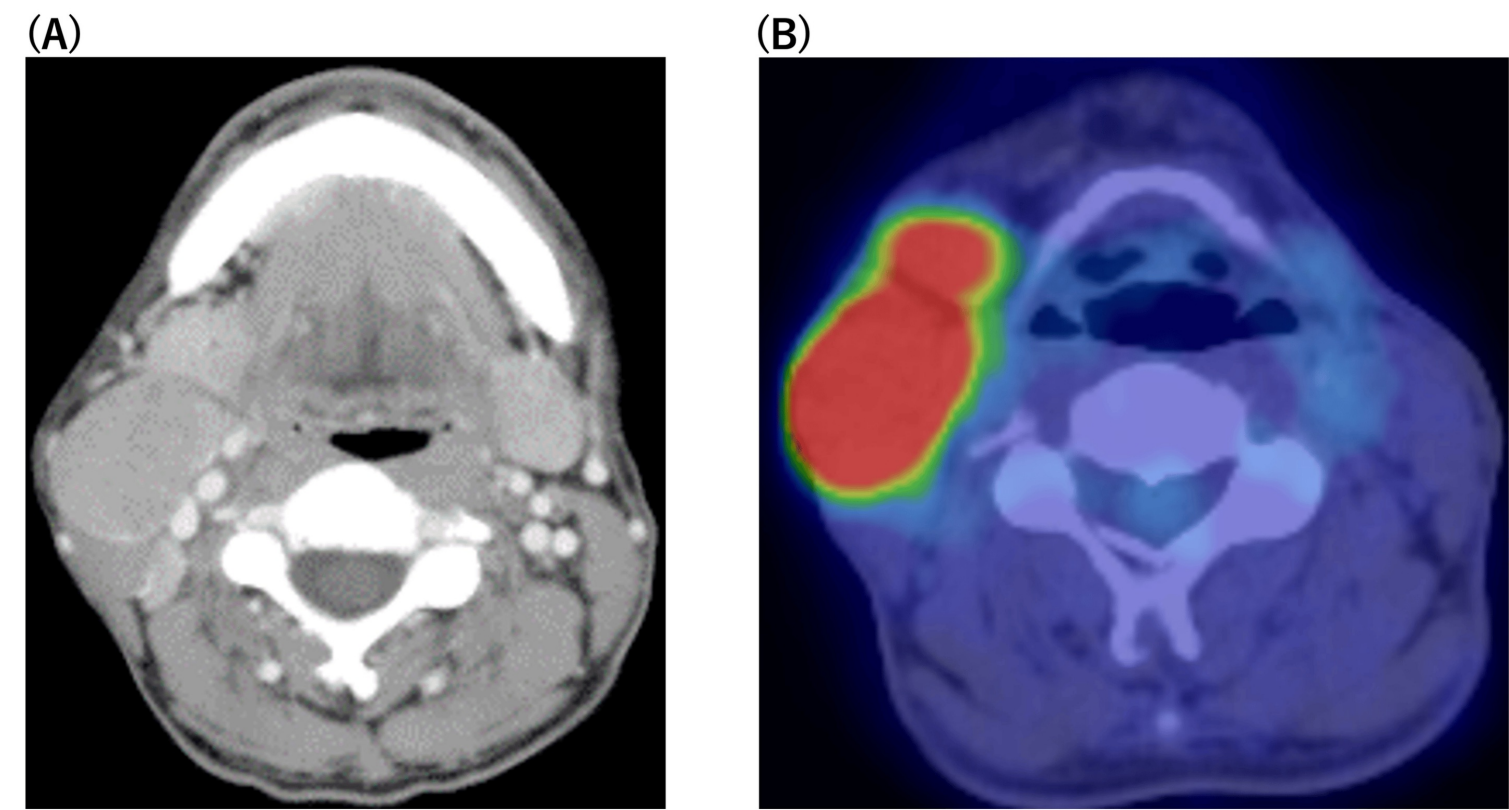
Pre-therapeutic findings. (A) Neck contrast-enhanced CT image showing swelling in the right submandibular gland with lymphadenopathy; (B) 18F fluorodeoxyglucose (FDG) PET-CT image showing high uptake of FDG in the right submandibular gland and lymph nodes.

A 53-year-old man was referred to our hospital with a right submandibular mass. Physical examination revealed two elastic hard masses in the right submandibular region, one measuring 3 cm and the other measuring 5 cm. Neck contrast-enhanced computed tomography (CECT) revealed the swelling of the right submandibular gland with lymphadenopathy in the right submandibular lesion (Figure 1A). Moreover, 18F fluorodeoxyglucose (FDG) positron emission tomography (PET)-CT confirmed a strong accumulation of FDG in the right submandibular gland and lymph nodes (Figure 1B).

**Figure 2:**
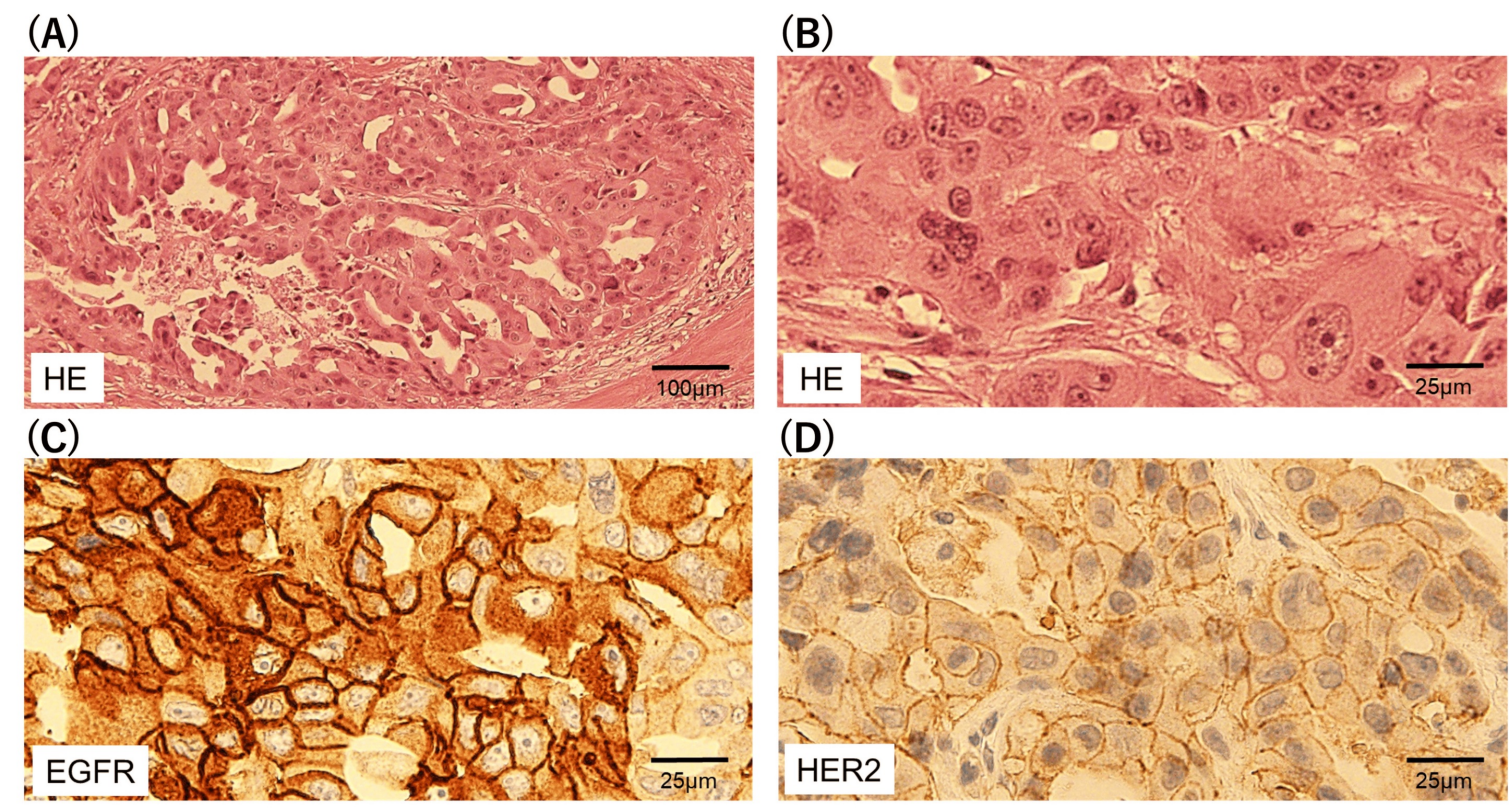
Immunohistochemical staining of the primary lesion. (A) Hematoxylin and eosin staining, 100 μm; (B) Hematoxylin and eosin staining, 25 μm; (C) EGFR staining; (D) HER2 staining. EGFR: epidermal growth factor receptor; HER2: human epidermal growth factor receptor 2

No evidence of distant metastasis was observed on these imaging findings. Malignant epithelial cells were identified using fine-needle aspiration cytology. Primary tumor resection and ipsilateral radical neck dissection were performed. The histopathological examination of the primary lesion revealed that the tumor cells exhibited pleomorphic nuclei, forming cribriform glands (Figures 2A, 2B). Immunohistochemical findings revealed that these tumor cells were highly and weakly positive for EGFR and HER2 expression, respectively (Figures 2C, 2D). Membrane staining intensity for EGFR and HER2 was scored as follows: no staining (0), weak (1+), intermediate (2+), and strong (3+). The immunostaining score (H-score) was calculated, ranging 0-300, using the following formula: 1×(% of 1+ cells)+2×(% of 2+ cells)+3×(% of 3+ cells). H-score of EGFR and HER2 were 270 and 90, respectively.

Enlarged lymph nodes exhibited similar histopathological findings. The patient was diagnosed with right SDC (cT2N2aM0, stage IVA), and adjuvant radiotherapy (50 Gy) was administered.

**Figure 3:**
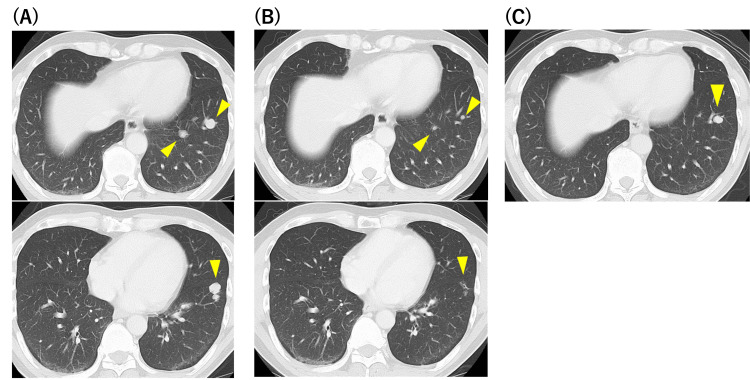
Chest CT images revealed the clinical course of pulmonary metastases. (A) Chest CT revealed multiple pulmonary metastases five months after the surgery; (B) Multiple pulmonary metastases shrank with chemotherapy; (C) Single pulmonary nodule appeared 15 months after the chemotherapy.

Nearly five months after the surgery, multiple pulmonary metastases were observed in the chest CT scan (Figure 3A). As the expression of EGFR was highly positive in the primary tumor, the patient received 12 cycles of weekly chemotherapy with cetuximab (first administration: 400 mg/m^2^; second and thereafter: 250 mg/m^2^) and paclitaxel (80 mg/m^2^). Most of the pulmonary metastases disappeared (Figure 3B), and the chemotherapy was terminated owing to adverse dermatological effects. Fifteen months after chemotherapy, a single pulmonary nodule was found to be enlarged in chest CT (Figure 3C).

**Figure 4:**
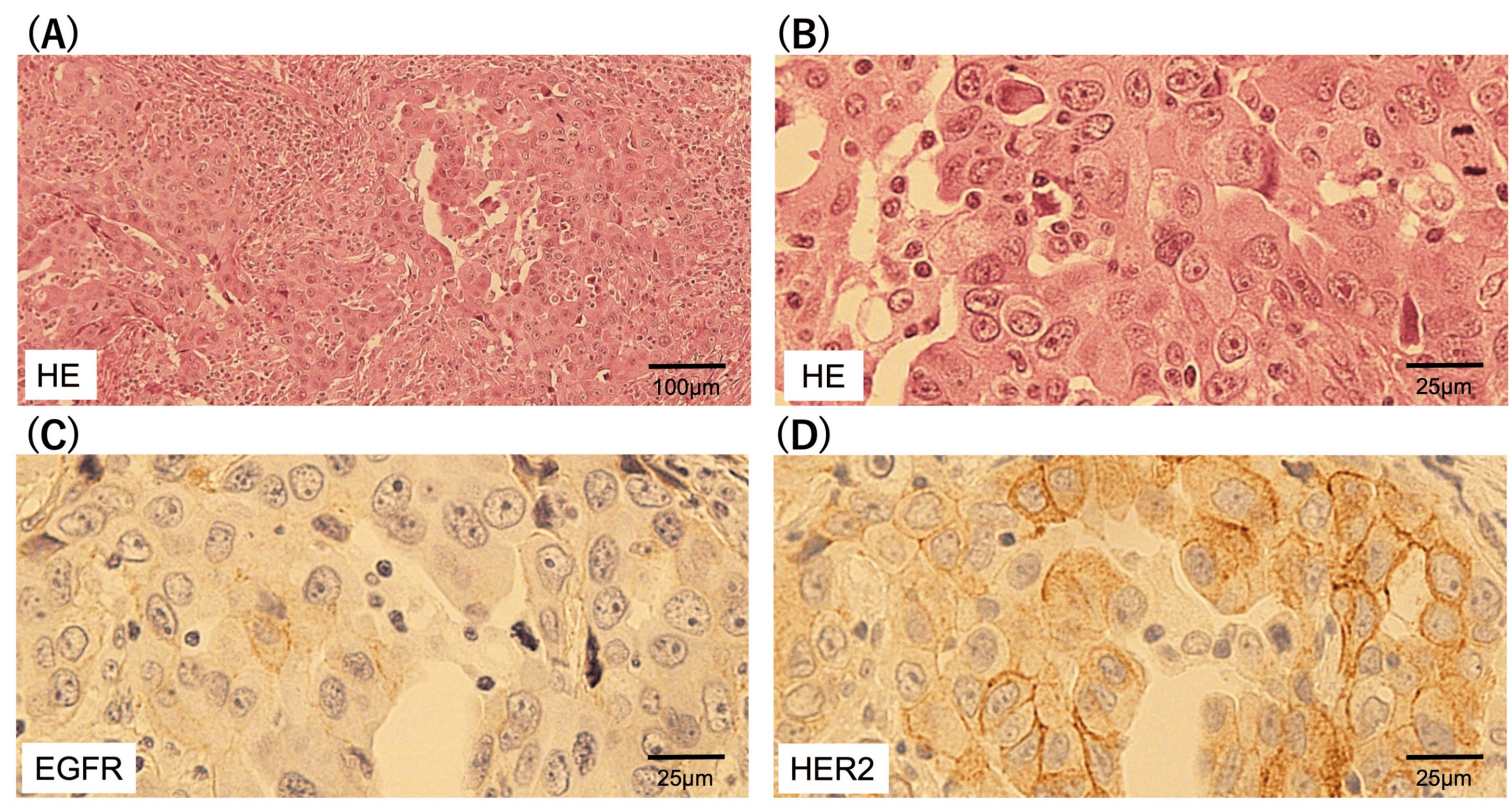
Immunohistochemical staining of pulmonary metastases. (A) Hematoxylin and eosin staining, 100 μm; (B) Hematoxylin and eosin staining, 25 μm; (C) EGFR staining; (D) HER2 staining EGFR: epidermal growth factor receptor; HER2: human epidermal growth factor receptor 2

The resection of the pulmonary tumor was performed, and the tumor was confirmed as the metastasis of SDC, which exhibited hematoxylin and eosin staining characteristics similar to those of primary SDC (Figures 4A, 4B). The primary and metastatic samples were reviewed by the same pathologists. Although the weak positivity of HER2 expression was maintained, the expression of EGFR was markedly downregulated in the metastatic tumors compared with that in the primary tumors (Figures 4C, 4D). H-score of EGFR and HER2 were 10 and 130, respectively.

The patient has remained alive without any additional treatments for seven years after pulmonary metastasectomy, with no evidence of recurrence.

## Discussion

SDC is an aggressive histological type of cancer that accounts for 5-10% of salivary gland cancer. Kusafuka et al. reported a five-year survival rate of approximately 50% with a high incidence of distant post-operative metastases after surgical resection [1]. As most SDCs occur in the parotid gland, the involvement of the submandibular gland is relatively rare, accounting for less than 20% of SDCs [1]. The prognosis of SDC in the submandibular gland is poorer than that in the parotid gland because of a high local recurrence rate of approximately 66% [10]. Hitherto, a standard treatment for SDC has not been defined because of its rare histology. Margin-free surgical resection with post-operative radiotherapy is frequently performed to achieve local control; however, post-operative radiotherapy does not improve the overall prognosis of SDC [11]. Extracapsular spread and vascular and lymphatic invasion, but not poorly differentiated clusters, nuclear polymorphism, or mitotic index, are negative prognostic markers [12,13]. Although chemotherapy is considered for end-stage or disseminated SDC, no regimen has been recommended.

The unique biology of SDC sheds light on the development of novel treatments. HER2 and AR expressed in SDC may be targets for molecule-targeted therapy. Several reports have suggested that androgen deprivation therapy can increase the survival of patients with SDC, and a randomized phase II trial is ongoing (DUCT study) [14]. The survival benefit of anti-HER2 antibodies in combination with chemotherapy has been observed in several phase II studies [15]. In patients with dual HER2- and AR-positive SDC, HER2-targeted therapy resulted in better overall survival than when using androgen deprivation therapy as first-line therapy [15]. The expression of EGFR is widely observed in salivary gland cancer regardless of the type of tumor [4], and it is expressed in 24-55% cases of SDC [4,5], comparable to that of HER2 [6]. According to the literature, an anti-EGFR antibody therapy combined with chemotherapy effectively controls SDC [8,16]. In the present case, the use of a multidisciplinary approach, including anti-EGFR antibody therapy and pulmonary metastasectomy, enabled the patient to maintain complete remission for more than seven years, indicating that cetuximab could be an option for SDC treatment in addition to anti-HER2 antibody therapy and androgen deprivation therapy.

As the survival of patients with SDC is not dependent on the positivity of EGFR or HER2 expression [17], these growth factor receptors may not be essential for SDC proliferation. As a proof of concept, the expression of EGFR was diminished in pulmonary metastases that escaped cetuximab-induced tumor cytotoxicity in this study. The loss of target molecules in a tumor population is a mode of escape from molecule-targeted therapy. Anti-HER2 therapy suppresses HER2 expression in endometrial cancer [18]. As the downregulation of EGFR with anti-EGFR antibody therapy has not been reported, we provided evidence that cetuximab could select tumors with suppressed EGFR expression. Thus, several candidate molecules must be identified for molecule-targeted therapies in cases where the corresponding target is downregulated. In cases where only biopsy samples collected at the time of initial diagnosis are available, pathological examination using these samples may not reflect the biology of the recurrent tumor. It is recommended that the latest sample be examined for the expression of the target molecule. Metastasectomy is an option for the treatment of pulmonary oligometastases in the absence of targetable molecules. In the present case, pulmonary metastasectomy helped in completely removing the cetuximab-resistant tumor with a favorable prognosis. In addition to adenoid cystic cancer [19], pulmonary metastasectomy is a feasible approach for the treatment of the oligometastasis of SDC, particularly if complete resection is possible.

A limitation of this study is that, although the patient was HER2-positive, we were unable to offer chemotherapy with trastuzumab because of insurance issues. The use of trastuzumab for the treatment of patients with advanced or recurrent salivary gland cancer has been covered by insurance since 2021 in Japan, and an anti-HER2 antibody with chemotherapy is currently available to treat SDC with HER2 expression. If the expression of HER2 is downregulated by trastuzumab but that of EGFR is positive, cetuximab would be a promising treatment option. Another limitation is that it was difficult to determine whether the downregulation of EGFR expression was because of the selective pressure of cetuximab. As the frequency of genetic alterations in HER2 and BRAF is altered during metastasis in SDC [20], metastatic modifications in tumors, including epithelial-to-endothelial transition, may also impact the molecular biology of SDC. Transcriptomic and genomic analysis using pre- and post-cetuximab therapy samples is warranted to further evaluate the mechanism of EGFR downregulation. As this is a single case report, careful clinical judgement is necessary to apply the findings in the clinical practice.

## Conclusions

We encountered a case of primary SDC of the submandibular gland that remained in remission for more than seven years with multimodal therapies, including surgical resection, radiotherapy, cetuximab-based chemotherapy, and pulmonary metastasectomy. Cetuximab treatment effectively diminished most pulmonary metastases, and cetuximab-resistant tumors lost EGFR expression. Based on the clinical results of the present case, it can be concluded that selective pressures caused by molecule-targeted therapy can help downregulate the expression of the corresponding molecule, and pathological examinations must be performed using the latest tissue samples, if available.
